# GM-CSF increases LPS-induced production of proinflammatory mediators via upregulation of TLR4 and CD14 in murine microglia

**DOI:** 10.1186/1742-2094-9-268

**Published:** 2012-12-13

**Authors:** Bijay Parajuli, Yoshifumi Sonobe, Jun Kawanokuchi, Yukiko Doi, Mariko Noda, Hideyuki Takeuchi, Tetsuya Mizuno, Akio Suzumura

**Affiliations:** 1 Department of Neuroimmunology, Research Institute of Environmental Medicine, Nagoya University, Furo-cho, Chikusa-ku, Nagoya, 464-8601, Japan; 2 Department of Anatomy, School of Medicine, Keio University, Shinanomachi, Tokyo, Japan

**Keywords:** Microglia, TLR4, CD14, GM-CSF, NF-κB

## Abstract

**Background:**

Microglia are resident macrophage-like cells in the central nervous system (CNS) and cause innate immune responses via the LPS receptors, Toll-like receptor (TLR) 4 and CD14, in a variety of neuroinflammatory disorders including bacterial infection, Alzheimer’s disease, and amyotrophic lateral sclerosis. Granulocyte macrophage-colony stimulating factor (GM-CSF) activates microglia and induces inflammatory responses via binding to GM-CSF receptor complex composed of two different subunit GM-CSF receptor α (GM-CSFRα) and common β chain (βc). GM-CSF has been shown to be associated with neuroinflammatory responses in multiple sclerosis and Alzheimer’s disease. However, the mechanisms how GM-CSF promotes neuroinflammation still remain unclear.

**Methods:**

Microglia were stimulated with 20 ng/ml GM-CSF and the levels of TLR4 and CD14 expression were evaluated by RT-PCR and flowcytometry. LPS binding was analyzed by flowcytometry. GM-CSF receptor complex was analyzed by immunocytechemistry. The levels of IL-1β, IL-6 and TNF-α in culture supernatant of GM-CSF-stimulated microglia and NF-κB nuclear translocation were determined by ELISA. Production of nitric oxide (NO) was measured by the Griess method. The levels of p-ERK1/2, ERK1/2, p-p38 and p38 were assessed by Western blotting. Statistically significant differences between experimental groups were determined by one-way ANOVA followed by Tukey test for multiple comparisons.

**Results:**

GM-CSF receptor complex was expressed in microglia. GM-CSF enhanced TLR4 and CD14 expressions in microglia and subsequent LPS-binding to the cell surface. In addition, GM-CSF priming increased LPS-induced NF-κB nuclear translocation and production of IL-1β, IL-6, TNF-α and NO by microglia. GM-CSF upregulated the levels of p-ERK1/2 and p-p38, suggesting that induction of TLR4 and CD14 expression by GM-CSF was mediated through ERK1/2 and p38, respectively.

**Conclusions:**

These results suggest that GM-CSF upregulates TLR4 and CD14 expression in microglia through ERK1/2 and p38, respectively, and thus promotes the LPS receptor-mediated inflammation in the CNS.

## Background

Microglia are resident macrophage-like cells in the central nervous system (CNS). They become activated in response to bacterial infection including gram-negative bacteria, *Candida albicans*, and *Cryptococcus neoformans*, and initiate an inflammatory response [1,2]. The principal mechanism that initiates microglial response may be the toll-like receptor (TLR) family of receptors [1,2]. TLRs serve as pathogen-associated molecular pattern recognition receptors that bind microbial molecular motif with high affinity, and play a central role in the initiation of cellular innate immune responses [3]. TLR4 and CD14 are constitutively expressed in microglia [1,4] and their ligand, lipopolysaccharide (LPS) induces production of inflammatory mediators including TNF-α, IL-6, and nitric oxide (NO) [4,5], via the nuclear factor κB (NF-κB) signaling pathway [6].

It is reported that TLR4 on microglia recognizes fibrillar Aβ [7]. Activation of TLR4 is reported to be associated with increased production of TNF-α and IL-1β in the CNS of APPswe/PS1 transgenic mice, a mouse model of Alzheimer’s disease (AD) [8]. Genetic deficiency in CD14 reduces Aβ-induced microglial NO and IL-6 production [9], and Alzheimer pathology in APPswe/PS1 transgenic mice [10], suggesting that the LPS receptor plays an important role in inflammation in AD. Furthermore, human mutations in (Cu/Zn) superoxide dismutase-1 (mSOD1) G93A and G85R, which cause non-cell-autonomous motor neuron death in amyotrophic lateral sclerosis (ALS), activate microglia and induce production of inflammatory mediators, including TNF-α, IL-1β, and NO [11]. TLR4 expression in microglia is reportedly increased in the spinal cord of ALS patients [12]. Thus, these findings suggest that the LPS receptor plays an important role, not only in pathogen-induced inflammation, but also in inflammation caused by misfolded protein, like fibrillar Aβ and mSOD1 in neurodegenerative diseases.

Granulocyte macrophage colony-stimulating factor (GM-CSF) is a pleiotropic cytokine secreted by a wide variety of cells including endothelial cells, monocytes, astrocytes, and T cells [13-17]. GM-CSF exerts its biological function by binding to GM-CSF receptor complex composed of two different subunits of GM-CSF receptor alpha (GM-CSFRα) which shows ligand-specific binding, and a common β (βc) subunit, which is shared with closely associated cytokines IL-3 and IL-5, and acts as the signal transduction chain [18,19]. GM-CSF signal is transmitted through Janus kinase (JAK) 2, mitogen activated protein kinase (MAPK) and phosphatidylinositol 3-kinase (PI3K) [20-22].

GM-CSF has been shown to be upregulated in various neurological disorders like AD, vascular dementia, multiple sclerosis (MS) [23,24]. Indeed, GM-CSF-deficient mice are reportedly resistant to experimental autoimmune encephalomyelitis, an animal model of MS [16,17], in which microglial activation is reduced. Moreover, neutralization of GM-CSF by antibodies suppresses microglial activation in the cerebral cortices of the mouse model of AD, Tg2576 mice [25]. Thus, it is expected that GM-CSF is associated with promoting CNS inflammation via microglial activation. We previously reported that GM-CSF was able to induce phagocytosis, proliferation, IL-6 production, and MHC class II expression in microglia [26,27]. However, GM-CSF by itself failed to induce some proinflammatory mediators such as TNF-α and NO, suggesting that GM-CSF might promote inflammation in the CNS via activating microglia both directly and indirectly. However, the mechanisms by which GM-CSF enhances such a broad neuroinflammatory response are still far from clear.

Here, we show that the GM-CSF receptor complex, GM-CSFRα and GM-CSFRβ, is strongly expressed in microglia. GM-CSF enhances TLR4 and CD14 expression in microglia, leading to increase of LPS-induced NF-κB nuclear translocation and increased LPS-mediated IL-6, TNF-α, and NO production. GM-CSF-induced expression of TLR4 and CD14 was mediated through ERK1/2 and p38, respectively. These results suggest that GM-CSF promotes the LPS receptor-mediated neuroinflammation via upregulating TLR4 and CD14 expression in microglia.

## Material and methods

### Reagents

Recombinant mouse GM-CSF was obtained from R&D systems (Minneapolis, MN, USA). LPS was obtained from Sigma-Aldrich (St Louis, MO, USA). Wortmannin (PI3K inhibitor), U0126 (MEK1/2 inhibitor), SB203580 (p38 inhibitor), SP600125 (c-Jun N-terminal kinase (JNK) inhibitor), and NF-κB inhibitor SN50 were obtained from Calbiochem (Gibbstown, NJ, USA). Anti-phospho-p44/42 MAPK (p-ERK1/2), anti-p42/44 MAPK antibodies (extracellular signal-regulated kinase 1/2: ERK1/2), anti-phospho-p38 and anti-p38 were obtained from Cell Signaling Technology (Danvers, MA, USA). The other antibodies used were as follows: anti-TLR4 (eBioscience, San Diego, CA, USA), anti-CD14 (BioLegend, San Diego, CA, USA), anti-CD11b (Serotec, Kidlington, UK), anti-GFAP (Dako, Glostrup, Denmark), anti-Iba1 (Wako, Osaka, Japan), anti-MAP2 (Merck Millipore, Billerica, MA, USA), anti-GM-CSFRα (R&D systems, Minneapolis, MN, USA), and anti-NeuN (Merck Millipore).

### Cell culture

All animal experiments were conducted under protocols that were approved by the Animal Experiment Committee of Nagoya University. All primary cultures were prepared from C57BL/6 mice (Japan SLC, Hamamatsu, Japan).

Microglia were isolated from primary mixed glial cell cultures prepared from newborn mice on day 14 using the shaking-off method as previously described [27]. The purity of the cultures (> 99%) was determined by anti-CD11b immunostaining. The cultures were maintained in Dulbecco’s modified Eagle’s minimum essential medium (Sigma-Aldrich) supplemented with 10% fetal bovine serum (SAFC Biosciences, Lenexa, KS, USA), 5 μg/ml bovine insulin (Sigma-Aldrich), and 0.2% glucose.

Primary neuronal cultures were prepared from the cortices of embryos at embryonic day 17 (E17) as described previously [28]. Briefly, cortical fragments were dissociated into single cells in dissociation solution (Sumitomo Bakelite, Akita, Japan) and resuspended in nerve culture medium (Sumitomo Bakelite). Neurons were seeded onto 12-mm polyethyleneimine-(PEI)-coated glass cover slips (Asahi Techno Glass Corp, Chiba, Japan) at a density of 5 × 104 cells/well in 24-well plates. The purity of the culture was more than 95% as determined by NeuN-specific immunostaining.

Primary astrocytes were isolated as previously described [29]. Briefly, microglial cells were removed from mixed glial cell cultures from newborn mice and the remaining cultures were trypsinized and re-plated in tissue culture plates. Cultures after three passages were used as astrocytes. The purity of the culture was more than 95% as determined by glial fibrillary acidic protein (GFAP)-specific immunostaining.

### Reverse transcription (RT)-PCR

For quantitative PCR, microglia were treated with GM-CSF (20 ng/ml) for varying times (6 to 72 h). Similarly, microglia were treated with GM-CSF (20 ng/ml) for 48 h or left untreated in RepCell® (CellSeed, London, UK), in which cells are adherent at 37°C but detach from the plate at 20 to 25°C. Then the cells were harvested, washed twice and seeded on 24-well plates at a density of 1 × 10^5^ cells/well. After stimulation with varying concentrations of LPS for 48 h, the total cellular RNA was extracted using the RNeasy Mini Kit (Qiagen, Hilden, Germany). cDNA was synthesized from total cellular RNA that was denatured for 5 minutes at 65°C, followed by reverse transcription reaction using the SuperScript II (Life Technologies, Carlsbad, CA, USA). The cDNA served as a template to amplify genes in RT-PCRs with TaqMan Gene Expression assays (Applied Biosystems), Universal PCR Master Mix (Applied Biosystems), and Rotor-Gene Q (Qiagen). Expression levels of target genes were calculated using a comparative method and normalized to *GAPDH* expression levels as previously described [30]. The following primers and probes were obtained from Applied Biosystems: IL-1β, Mm00434228_m1; IL-6, Mm00446190_m1; TNF-α, Mm00443258_m1; *GAPDH*, Mm99999915_g1; NOS2, Mm00440502_m1; CD14, Mm00438094_m1; and TLR4, Mm00445273_m1.

For semi-quantitative PCR, the total cellular RNA from astrocytes, neurons and microglia were extracted using the RNeasy Mini Kit (Qiagen). cDNA was synthesized from total cellular RNA as described above with SuperScript II (Life Technologies) and semiquantitative PCR was performed using Ampli Taq DNA polymerase (Applied Biosystems) as previously described [31] using the following the specific primer sets: GAPDH GAPDH sense, 5^′^–ACTCACGGGAAATTCAACG–3^′^; GAPDH antisense, 5^′^–CCCTGTTGCTGTAGCCGTA–3^′^; GM-CSFRα sense, 5^′^–TGGCGAACGACTTGTCACTGCT–3^′^; GM-CSFRα antisense, 5^′^–GCACCTTGACCTTGTGACCT–3^′^; GM-CSFRβ sense, 5^′^–TGTTCCAGGATGGAGGTAAA–3^′^; GM-CSFRβ an-tisense, 5^′^–CCCACACTGCACATCCATAG–3^′^.

### Flow cytometry (FCM)

FCM was done as previously described [26]. Briefly, microglia cells treated with GM-CSF or left untreated were blocked with Fc block for 30 minutes in fluorescence-activated cell sorter (FACS) buffer and then cells were stained with phycoerythrin (PE)-conjugated-anti-TLR4, PE-conjugated-anti-CD14, or isotype-matched control. The cells were subsequently analyzed using a Cytomics FC500 (Beckman Coulter, Brea, CA, USA). To assess LPS binding, microglia cultured in the presence or absence of GM-CSF for 48 h, were treated with Alexa 488-conjugated LPS for 1 h. After washing twice to remove any unbound LPS, cells were analyzed using a Cytomics FC500.

### Immunocytochemistry

Immunocytochemistry was done as previously described [32]. Microglia, astrocytes or neurons plated on glass cover slips were fixed with 4% paraformaldehyde for 10 minutes, cells were then permeabilized with 0.05% Triton X-100 for 5 minutes and blocked with 5% goat serum for 1 h, followed by incubation with anti-iba1, anti-GFAP, or anti-MAP2 and GM-CSFRα, antibodies overnight at 4°C. Then, the cells were incubated with Alexa 488- or Alexa 568-conjugated secondary antibodies for 1 h. Cells were examined with a deconvolution fluorescence microscope system (Bio Zero, Keyence, Osaka, Japan).

### Measurement of NO and cytokines

Microglia were treated with GM-CSF for 48 h or left untreated in RepCell. Then the cells were harvested, washed twice and seeded at a density of 1 × 10^5^ cells/well in 24 well plates. Then the cells were treated with varying concentrations of LPS (0.01 to 1 μg/ml) for 48 h. Supernatants were collected and NO production was determined by the Griess reaction as previously described [31]. The levels of IL-1βIL-6 and TNF-α in culture supernatant were determined by ELISA according to the manufacturer’s instructions (BD Biosciences).

### NF-κB nuclear translocation

Microglia primed with GM-CSF for 48 h or left untreated were seeded on 6-well plates at 1 × 10^7^ cells/well and treated with LPS (1 μg/ml) for 0 to 30 minutes. Nuclear fractions from the cells were separated and the levels of NF-κB in the nuclear fractions were analyzed using the NF-κB/p65-active ELISA kit (Imgenex, San Diego, CA, USA) according to the manufacturer’s instructions.

### Western blotting

Microglia were treated with GM-CSF (20 ng/ml) for varying times (15 to 120 minutes). After washing with PBS, cells were lysed with TNES buffer (1 M Tris–HCl, 20% SDS and 2.5% glycerol) containing phosphatase (Sigma-Aldrich) and protease inhibitor (Roche, Mannheim, Germany); 50 μg of protein from the total lysate was assayed for phosphorylated ERK1/2, phosphorylated p38, total ERK and total p38 by western blotting as described previously [31].

### Statistical analysis

Statistically significant differences between experimental groups were determined by one-way analysis of variance (ANOVA) followed by Tukey test for multiple comparisons. Statistical analysis was performed using the software program Prism 4.0 (GraphPad Software, San Diego, CA, USA). *P*-values less than 0.05 were considered statistically significant.

## Results

### GM-CSFR complex is expressed in microglia

**Figure 1:**
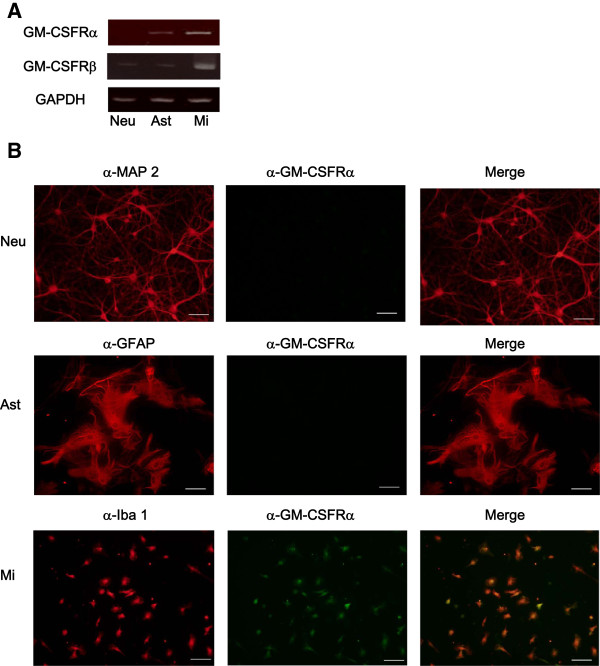
**Granulocyte macrophage-colony stimulating factor (GM-CSF) receptor complex is predominantly expressed in microglia in the central nervous system (CNS).** (**A**) The expression levels of GM-CSF Rα and GM-CSF Rβ mRNA in neurons, astrocytes, and microglia were analyzed by semi-quantitative PCR. (**B**) Surface expression of GM-CSF Rα in neurons, astrocytes and microglia was analyzed by immunocytochemistry. Data are representative of three independent experiments. Scale bar represents 50 μm.

We first assessed the basal expression of GM-CSFRα and GM-CSFRβ mRNA in microglia, astrocytes and neurons. Microglia expressed both GM-CSFRα and GM-CSFRβ mRNA (Figure 1A). The expression of GM-CSFRα and GM-CSFRβ mRNA in astrocytes and GM-CSFRβ mRNA in neurons was very weak (Figure 1A). Immunocytochemistry confirmed that microglia, but not neurons and atrocytes, expressed GM-CSFRα protein (Figure 1B). These results indicate that functional GM-CSFR is expressed in microglia in the CNS.

### GM-CSF increases TLR4 and CD14 expression in microglia

**Figure 2:**
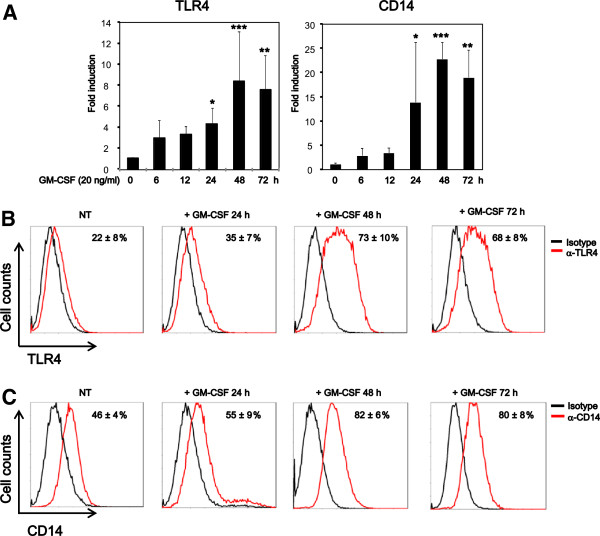
**Granulocyte macrophage-colony stimulating factor (GM-CSF) increases toll-like receptor (TLR) 4 and CD14 expression in microglia.** (**A**) Microglia were treated with GM-CSF (20 ng/ml) for 6 to 72 h and analyzed for expression of TLR4 and CD14 mRNA by real-time PCR. (**B**, **C**) Microglia treated with GM-CSF at 20 ng/ml were analyzed for surface expression of TLR4 and CD14 by flow cytometry (FCM). Data indicate mean ± SD of three independent experiments. *P < 0.05, **P < 0.01, ***P < 0.001 (GM-CSF-treated vs. -untreated).

**Figure 3:**
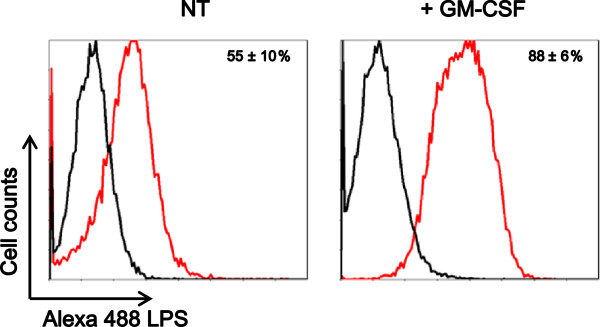
**Granulocyte macrophage-colony stimulating factor (GM-CSF) enhances lipopolyssacharide (LPS) binding to microglia.** Microglia were treated with GM-CSF at 20 ng/ml for 48 h and then treated with Alexa 488-conjugated LPS for 1 h. Cells were examined by flow cytometry (FCM). Data indicate mean ± SD of three independent experiments.

We next assessed expression of the LPS receptor, TLR4, and CD14 in microglia. GM-CSF time-dependently increased expression of TLR4 and CD14 mRNA, which peaked at 48 h and remained stabilized until 72 h in microglia (Figure 2A). FCM also showed that GM-CSF upregulated the surface expression of TLR4 and CD14 in microglia, which peaked at 48 h and remained stabilized until 72 h (Figure 2B and C). To further confirm that GM-CSF increased the LPS receptor expression, we assessed binding of Alexa488-conjugated LPS in GM-CSF-primed and -unprimed microglia. GM-CSF priming increased the surface binding of LPS (Figure 3). These results indicate that GM-CSF increase expression of TLR4 and CD14 in microglia.

### GM-CSF enhances LPS-induced production of inflammatory mediators

**Figure 4:**
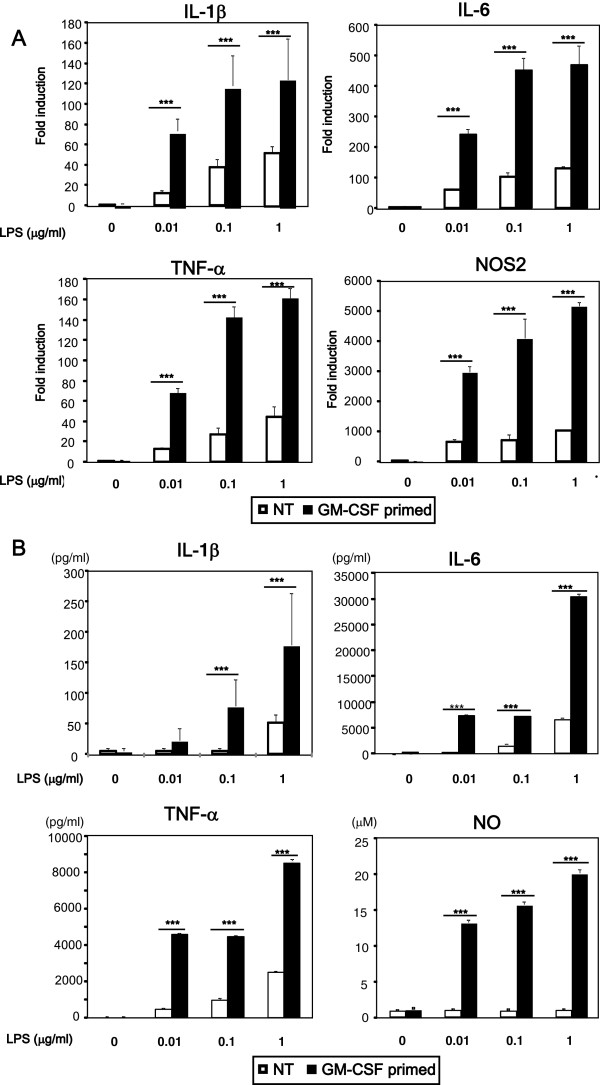
**Granulocyte macrophage-colony stimulating factor (GM-CSF) increases lipopolysaccharide (LPS)-induced IL-6, nitric oxide (NO), and TNF-α production.** After pretreating with GM-CSF or leaving untreated for 48 h, microglia were harvested, washed twice, and seeded at 1 × 10^5^ cells/well. Then the cells were treated with varying doses of LPS (0.01 to 1 μg/ml) for 48 h. (**A**) The expression levels of IL-1β, IL-6, TNF-α and NOS2, mRNA were assessed by quantitative real-time PCR. (**B**) Concentrations of IL-1β, IL-6, TNF-α , and NO in culture supernatant were analyzed by ELISA and the Griess method. Data indicate mean ± SD. Data are representative of three independent experiments. ***P < 0.001 (GM-CSF pretreated vs. untreated).

We then examined whether GM-CSF affects LPS-mediated function in microglia. We pretreated microglia with GM-CSF for 48 h, then the cells were harvested, washed, seeded at a density of 1 × 10^5^ cells/well in 24-well plates, and further stimulated with varying doses of LPS (0.01 to 1 μg/ml) for 48 h. GM-CSF priming increased LPS-induced expression of IL-1β, IL-6, TNF-α and NOS2 mRNA as compared to unprimed microglia (Figure 4A). Similarly, GM-CSF priming increased LPS-induced secretion of IL-1β, IL-6, TNF-α and NO (Figure 4B).

**Figure 5:**
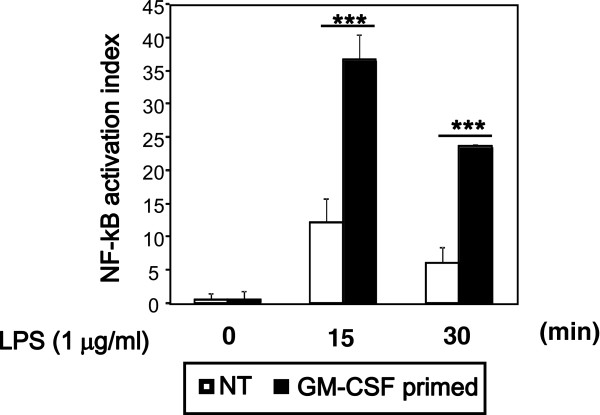
**Granulocyte macrophage-colony stimulating factor (GM-CSF) enhances lipolysaccharide (LPS)-induced nuclear factor (NF)-κB nuclear translocation.** After pretreating with GM-CSF or leaving untreated for 48 h, microglia were harvested, washed twice, and seeded at a density of 1 × 10^7^ cells/well. Then the cells were treated with LPS (1 μg/ml) for varying times (0 to 30 minutes). Nuclear fractions from the cells were separated and the levels of NF-kB/p65 in the nuclear fractions were assessed by ELISA. Data are representative of three independent experiments. ***P < 0.001 (GM-CSF-pretreated vs. -untreated).

**Figure 6:**
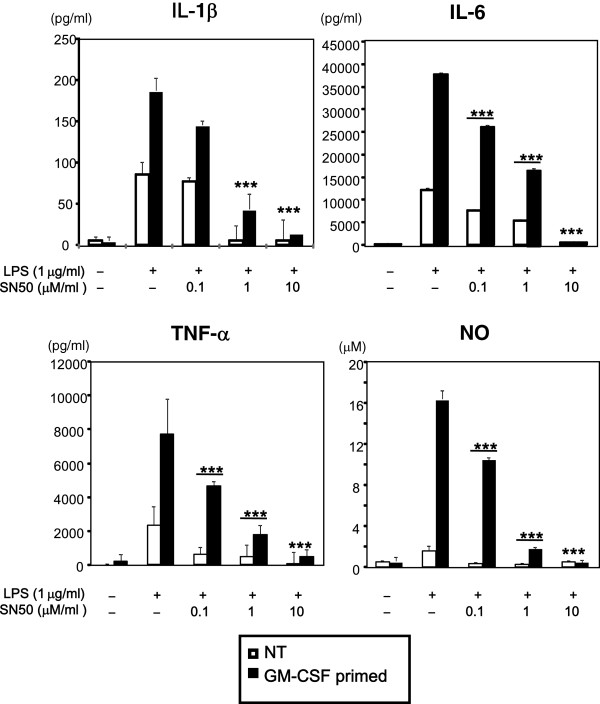
**SN50 decreases lipolysaccharide (LPS)-induced production of IL-6, nitric oxide (NO), TNF-α both in granulocyte macrophage-colony stimulating factor (GM-CSF)-treated and -untreated microglia.** After pretreating with GM-CSF or leaving untreated for 48 h, microglia were harvested, washed twice, and seeded at 1 × 10^5^ cells/well. Then the cells were treated SN50 for 1 h before stimulation with LPS at 1 μg/ml for 48 h. Concentrations of IL-1β, IL-6, TNF-α and NO were determined by ELISA and the Griess method. Data indicate mean ± SD. Data are representative of three independent experiments. ***P < 0.001 (untreated and SN50-treated).

NF-κB plays a critical role in the production of various proinflammatory molecules, including IL-6 and TNF-α, by activation of TLR4 [6]. To further characterize GM-CSF priming of LPS response, to find out whether increased TLR4 and CD14 are associated with enhanced nuclear translocation of NF-kB, we assessed nuclear translocation of NF-kB. GM-CSF priming significantly increased the levels of LPS-induced NF-κB nuclear translocation as compared to unprimed cells (Figure 5). To reveal the requirement of NF-κB in LPS-induced increased production of cytokines in GM-CSF-primed or -unprimed cells, we inhibited NF-κB signaling with SN50 before LPS stimulation, and assessed IL-1β, IL-6, TNF-α and NO production by ELISA and Griess reagent, respectively. SN50 dose-dependently inhibited production of LPS-induced IL-1β, IL-6, TNF-α and NO in both unprimed and GM-CSF-primed microglia, although a higher dose was required to completely inhibit the production of IL-1β, IL-6, TNF-α, and NO in GM-CSF-primed microglia (Figure 6). These results indicate that GM-CSF priming increases LPS-mediated production of IL-1β, IL-6, TNF-α and NO via increasing activation of NF-κB.

### GM-CSF-induced expression of TLR4 and CD14 is mediated via ERK1/2 and p38 activation, respectively

**Figure 7:**
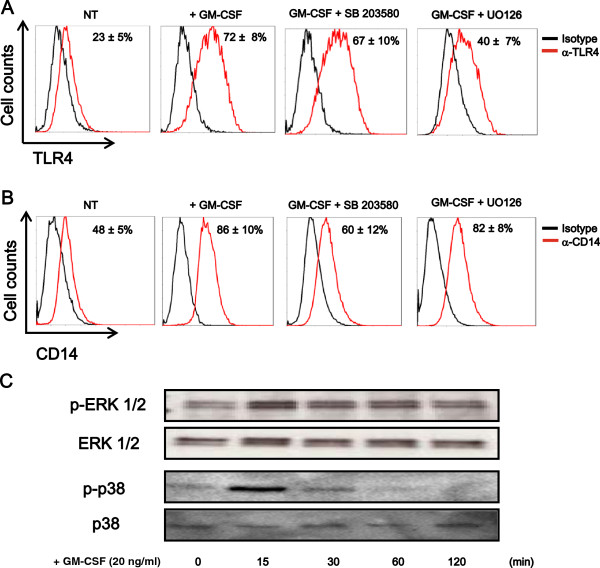
**Granulocyte macrophage-colony stimulating factor (GM-CSF) upregulates toll-like receptor (TLR) 4 and CD14 expression via extracellular signal-regulated kinase (ERK) 1/2 and p38, respectively.** (**A, B**) Microglia were pretreated with U0126 or SB 203580 for 1 h before stimulation with 20 ng/ml GM-CSF for 48 h. Surface expression levels of TLR4 were assessed by flow cytometry (FCM) (**A**). Data indicate mean ± SD of three independent experiments; *P* < 0.001 (between GM-CSF and GM-CSF + U0126), *P* < 0.05 (between NT and GM-CSF + U0126). Surface expression levels of CD14 were assessed by FCM (**B**). Data indicate mean ± SD of three independent experiments; *P* < 0.001 (between GM-CSF and GM-CSF + SB 203580); *P* > 0.05 (between NT and GM-CSF + SB 203580). (**C**) Microglia were treated with 20 ng/ml GM-CSF. Then cells were lysed and total cell lysates were analyzed by western blotting for p-ERK1/2, ERK1/2, p-p38, and p38. Data are representative of two independent experiments.

**Figure 8:**
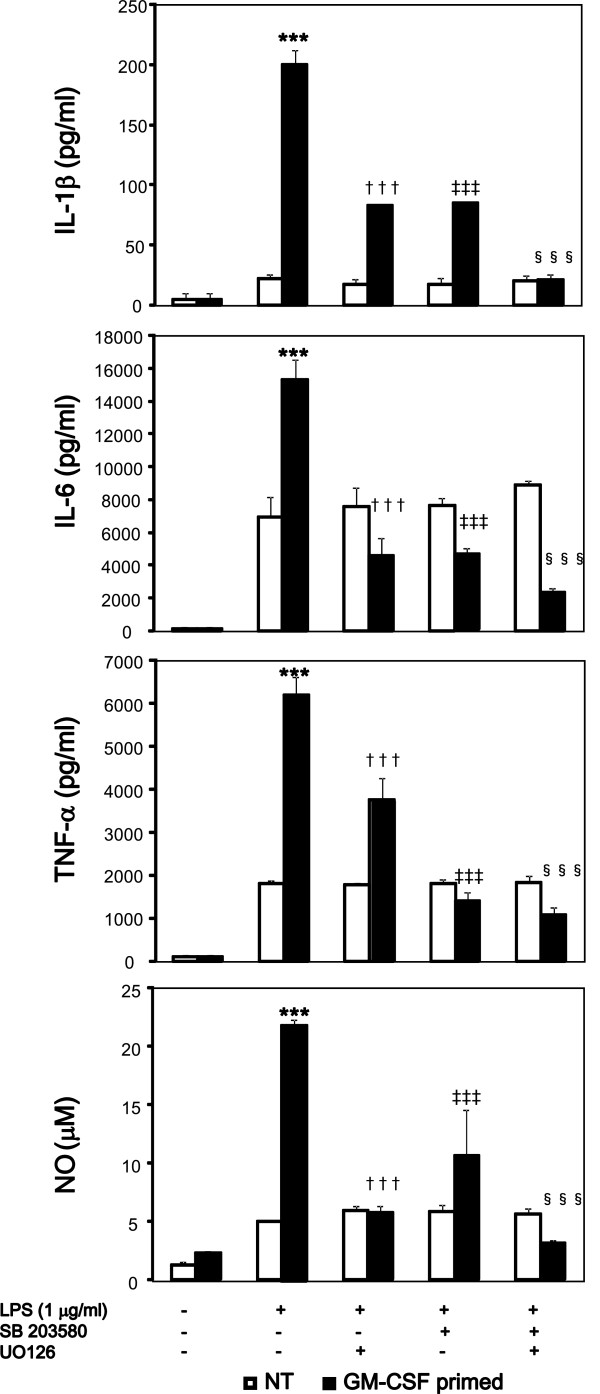
**Inhibition of extracellular signal-regulated kinase (ERK) 1/2, p38 decreases lipopolysaccharide (LPS)-induced production of IL-1β, IL-6, TNF-α and nitric oxide (NO) in granulocyte macrophage-colony stimulating factor (GM-CSF)-treated microglia.** Microglia were pretreated with U0126, SB 203580, or both, for 1 h and stimulated with GM-CSF (20 ng/ml) or left untreated for 48 h. Then the cells were harvested, washed twice, and seeded at a density of 1 × 10^5^ cells/well in 24-well plates. Cells were further stimulated with LPS (1 μg/ml) for 48 h. Concentrations of IL-1β, IL-6, TNF-α and NO were determined by ELISA and the Griess method respectively. Data indicate mean ± SD. Data are representative of two independent experiments. ***P < 0.001 (between GM-CSF-primed and -unprimed), ^†††^*P* < 0.001 (between GM-CSF-primed and SB 203580-pretreated), ^‡‡‡^*P* < 0.001 (between GM-CSF-primed and U0126-pretreated), ^§§§^*P* < 0.001 (between GM-CSF-primed and SB 203580 + U0126-pretreated).

GM-CSF has been shown to activate JAK2, PI3K, ERK1/2, and p38 in microglia [20-22]. We evaluated the pathway involved in GM-CSF-induced upregulation of TLR4 and CD14 expression in microglia. JAK2 inhibitor, PI3K inhibitor, Wortmannin, and NF-κB inhibitor, SN50 had no effect in GM-CSF-induced increase of TLR4 and CD14 expression. The MEK1/2 inhibitor, U0126 (1 μM/ml) suppressed GM-CSF-induced surface expression of TLR4 but did not reach basal level (Figure 7A). SB203580 had no effect in the surface expression TLR4. In contrast, the p38 inhibitor, SB203580 (1 μM/ml) suppressed GM-CSF-induced surface expression of CD14 to basal level (Figure 7B). U0126 had no effect in the surface expression of CD14. We further assessed the phosphorylation of ERK1/2 and p38 by western blotting. GM-CSF increased the levels of phosphorylated ERK1/2 and p38, respectively, which peaked at 15 minutes (Figure 7C). Accordingly, U0126, SB203580, or U0126 + SB203580 pretreatment before GM-CSF priming decreased LPS-mediated production of IL-1β, IL-6, TNF-α and NO in microglia (Figure 8). Taken together, these data indicate that GM-CSF increases TLR4 surface expression via ERK1/2 and that of CD14 via p38.

## Discussion

Here we have shown that GM-CSFR complex is expressed by microglia but not astrocytes and cortical neurons in the CNS, as no immunoreactivity for GM-CSFRα, which is required for binding GM-CSF, was found in GFAP-positive astrocytes or in MAP2-positive cortical neurons. In contrast to our finding, previous reports suggested expression of GM-CSFRα in astrocytes and neurons [33-35]. However, we could not find any staining in neurons and astrocytes. However, we cannot rule out the possibility of regional- or maturation-specific expression of GM-CSFRα in neurons, as described previously, and upregulation of GM-CSFRα in activated astrocytes under host-specific conditions.

We showed that GM-CSF increased both mRNA and surface expression of TLR4 and CD14 via activation of ERK1/2 and p38, respectively, and pretreatment with U0126, SB203580, or both, indeed inhibit LPS-induced production of proinflammatory mediators in the microglia incubated with GM-CSF. In the peripheral immune system, it has been shown that peritoneal macrophages from GM-CSF knockout mice are hyposensitive to LPS [36], and GM-CSF is required for the expression of TLR4 and CD14 in alveolar macrophages [37]. Similarly, we showed that GM-CSF enhances LPS-induced production of inflammatory mediators by promoting TLR4 expression, which is consistent with a previous report showing that the magnitude of LPS response correlates with total TLR4 expression levels [38]. Moreover, *Helicobacter pylori* LPS upregulate TLR4 expression via ERK1/2 in the gastric epithelial cell line, MKN28, and GM-CSF reportedly upregulate TLR4 expression via ERK1/2 in human blood monocytes respectively [39,40]. GM-CSF is reported to be required for CD14 expression in murine alveolar macrophages and human myeloid leukemia cells, whereas in human blood monocytes GM-CSF has been found to have no effect in CD14 expression [37,40,41]. Similarly, in the human monocytic cell line U937, both p38 and ERK1/2 activation have been shown to increase CD14 expression [42]. However, in our experiment, inhibiting p38 but not ERK1/2 decreased the surface expression of CD14. Thus, CD14 may be differentially regulated in tissue-resident monocyte/macrophage cell-like microglia, alveolar macrophage and circulating monocytes.

TLR4 is required for the initiation of innate immune responses to LPS [5,6]. CD14 is a key LPS co-receptor, pivotal in the initial binding of LPS and transfer of LPS to MD2/TLR4 complex to initiate signal cascades [43-46]. In addition to LPS, various endogenous molecules such as HMGB1 and HSP60, which are released from dying cells, and misfolded proteins like mutant SOD1 and fibriller Aβ, as well as fibronection, have been shown to bind to TLR4 and CD14 [7,11,47-50]. Surface expression of TLR4 and CD14 in microglia has been shown to be elevated in various neurological disorders like ALS and AD [12,51]. Indeed, chronic activation of TLR4 and CD14 by LPS has been shown to exacerbate disease in the mouse model of ALS [52]. It has also been shown that extracellular mSOD1 interacts with CD14 in microglia, which then associates with TLR2 and TLR4 activating the proinflammatory cascade [11]. Similarly, Aβ has been shown to bind both TLR4 and CD14 and induce inflammation [7-10]. Thus, GM-CSF may function to exacerbate ALS and AD by increasing TLR4 and CD14 expression in microglia. Supporting this hypothesis, GM-CSF knockout mice have been reported to have delayed onset and increased life span in the mouse model of ALS [53]. In addition, blocking GM-CSF with anti-GM-CSF antibodies reportedly decreased microglial activation in the mouse model of AD [25,54]. However, there are several conflicting reports. GM-CSF is reported to be beneficial in the mouse model of Parkinson’s disease and in stroke [55,56]. Thus, it is possible that GM-CSF may have both beneficial and detrimental effects, depending on the disease stages or pathogenesis. Further studies are needed to examine the role of GM-CSF in various neurological disorders.

## Conclusions

GM-CSF increases surface expression of TLR4 and CD14 in microglia, which is mediated by ERK1/2 and p38, respectively. Accordingly, GM-CSF upregulates LPS-induced IL-1β, IL-6, TNF-α, and NO production and may promote neuroinflammation.

## Abbreviations

AD: Alzheimer’s disease; ALS: Amyotrophic lateral sclerosis; ANOVA: One-way analysis of variance; βc: Common β chain; CNS: Central nervous system; ERK: Extracellular signal-regulated kinase; FACS: Fluorescence-activated cell sorter; FCM: Flow cytometry; GFAP: Glial fibrillary acidic protein; GM-CSF: Granulocyte macrophage-colony stimulating factor; IL: Interleukin; NOS2: Nitric oxide synthase 2; JAK: Janus kinase; LPS: Lipopolysaccharide; MAPK: Mitogen-activated protein kinase; mSOD: Mutations in superoxide dismutase; MS: Multiple sclerosis; NF-kB: Nuclear factor-κB; NO: Nitric oxide; PBS: Phosphate-buffered saline; PI3K: Phosphatidylinositol-3 kinase; PCR: Polymerase chain reaction; TLR: Toll-like receptor; TNF: Tumor necrosis factor.

## Competing interests

The authors declare that they have no competing interests.

## Authors’ contributions

BP performed the experiments and helped draft the manuscript. YD, JK, and NM performed the cell culture and were involved in the conception of study. TM and TH helped with the preparation of the manuscript. YS was involved in the conception and design of the study and helped draft the manuscript. AS was involved in the conception and design of the study and helped draft the manuscript. All authors read and approved the final manuscript.
